# Non-Deep Simple Morphophysiological Dormancy and Germination Characteristics of *Gentiana triflora* var. *japonica* (Kusn.) H. Hara (Gentianaceae), a Rare Perennial Herb in Korea

**DOI:** 10.3390/plants10101979

**Published:** 2021-09-22

**Authors:** Hyeon-Min Kim, Jun-Hyeok Kim, Da-Hyun Lee, Young-Ho Jung, Chung-Youl Park, Mi-Hyun Lee, Kyeong-Min Kim, Jae-Hyeon Lee, Chae-Sun Na

**Affiliations:** 1 Wild Plant Seed Research Division, Baekdudaegan National Arboretum, Bonghwa 36209, Korea; khm0766@koagi.or.kr (H.-M.K.); kjh9859@koagi.or.kr (J.-H.K.); dahyun0519@koagi.or.kr (D.-H.L.); jyh5250@koagi.or.kr (Y.-H.J.); doonas@koagi.or.kr (C.-Y.P.); hyun3176@koagi.or.kr (M.-H.L.); katty1502@koagi.or.kr (K.-M.K.); 2 Forest Bioresource Investigation Team, Baekdudaegan National Arboretum, Bonghwa 36209, Korea; ecojay@koagi.or.kr

**Keywords:** cold stratification, gibberellic acid, rare plant, seed propagation, underdeveloped embryos

## Abstract

This study investigated the kind of seed dormancy and seed germination of *Gentiana triflora* var. *japonica* (Kusn.) H. Hara for developing a seed propagation method. The seeds were collected in October 2020 from plants at Mt. Sobaeksan, Korea. In a water imbibition experiment, seed weights increased by >101.9% of their initial masses over 12 h. Effects of incubation temperature (5, 15, 20, 25, 15/6, or 25/15 °C), cold stratification period (5 °C; 0, 4, 8, or 12 weeks), and gibberellic acid (GA_3_; 0, 10, 100, or 1000 mg∙L^−1^) and potassium nitrate treatment (KNO_3_; 0, 1000, 2000, or 4000 mg∙L^−1^) on seed germination were investigated to characterize seed dormancy. These seeds exhibited underdeveloped embryos during seed dispersal. The seeds failed to reach the final germination of 15.0% after treatment at 5, 15, 20, 25, 15/6, or 25/15 °C. After cold stratification for 8 weeks, the germination increased dramatically by >90.0% compared to that at 0 weeks. After the GA_3_ treatment, the germination reached >80.0% within 5 days. The final germination was 90.0% in the 100 mg∙L^−1^ GA_3_ treatment group. However, the KNO_3_ treatment had no effect on seed germination. Therefore, the *G*. *triflora* var. *japonica* seeds exhibited non-deep simple morphophysiological dormancy.

## 1. Introduction

The success of seed germination is the most important feature for establishing and propagating plant seedlings in nature [1,2,3,4]. Several mechanisms control seed germination, including temperature, moisture, humidity, and light, among which seed dormancy is the most important [5]. Although seed dormancy is a critical plant trait from the ecological and conservation perspectives, understanding how to overcome seed dormancy can enable the use of wild plant species that are potentially valuable to the agricultural and horticultural industries [6,7]. Recently, consumers have become increasingly interested in medicinal plants and/or high-value crops using natural substances in the pharmaceutical, animal feed, human food, and cosmetics industries. In addition, plants, especially those with medicinal components, play an essential role in improving health care [8]. Numerous studies on the germination characteristics, seedlings, plant culture, and secondary metabolites of commonly grown potential medicinal plants, such as *Allium hirtifolium* Boiss., *Ocimum basilicum* L., *Crepidiastrum denticulatum*, *Salvia plebeian* R. Br., *Mesembryanthemum crystallinum* L., and *Oenanthe stolonifera* have been conducted [9,10,11,12,13,14]. Currently, the number of wild plants is decreasing due to climate change and the destruction of native habitats, and basic data on seed germination characteristics are required for forest restoration and conservation of these native plants. However, although various conservation and taxonomic studies on wild plants are being conducted, studies on mass propagation to industrialize these plants are insufficient. Therefore, it is necessary to not only examine seed germination to establish mass production, but to also investigate the seed dormancy type of wild plants to utilize their medicinal resources and restore forests.

Seed dormancy is classified by the developmental status of the embryo, water absorption capacity, and interrelationships of phytohormones in seeds. Baskin and Baskin [15] reported that seed dormancy is divided into five categories: physiological dormancy (PD), morphological dormancy (MD), morphophysiological dormancy (MPD), physical dormancy (PY), and combinational dormancy (PY + PD). Generally, the Gentianaceae family has been reported to exhibit PD or MPD [16]. Similarly, the seeds of the genus *Gentiana* display non-deep PD and intermediate–complex MPD [17,18,19]. Even among seeds of the same genus, the dormancy class is species-specific. Dormancy must be released in seeds with PD and MPD, and this requires seeds to be pretreated using cold (0–10 °C) and/or warm (≥15 °C) stratification, gibberellins (GAs), and ripening in dry storage [20]. Furthermore, MPD is classified into eight levels, and systematic studies are required to investigate the exact dormancy of seeds. In particular, gibberellic acid (GA_3_) is commonly used to break dormancy, enhance germination, promote embryo growth, and stimulate radicle emergence in many plant species [21,22,23,24]. In addition, GA_3_ is not only easy to apply to investigate seed dormancy release, but also does not require special equipment or facilities. GA_3_, however, does not enhance germination of all the species; in fact, germination may be suppressed depending on the method and/or concentration used. Similarly, cold and/or warm stratification are widely known to aid in releasing dormancy and promoting embryo growth of PD- or MPD-type seeds [20,25,26].

In Gentianaceae, the genus *Gentiana* includes approximately 360 species that are distributed worldwide, mainly in temperate, arctic, and alpine habitats of the northern hemisphere [27]. The genus *Gentiana* has been reported to include 16 taxa distributed throughout Korea; among them, *G*. *triflora* var. *japonica* (English name: East Asian clustered gentian, Korean name: Gwa-nam-pul) is a rare (least-concern, LC) perennial species in Korea [28]. Several taxonomic [29] and pharmacognostical studies [30] have been conducted on *Gentiana* plants in Korea. Therefore, it is necessary to investigate not only the in situ, but also the ex situ conservation. Studies have also identified that the *Gentiana* genus possesses the pharmacological ingredients gentiopicroside, gentiin, gentisin, gentiopicrin, and gentiotriose in its roots, and the plants are used as an Oriental medicine for anorexia, carditis, liver protection, cholangitis, and anticancer activity [31,32].

In this study, we investigated the response of *G*. *triflora* var. *japonica* seeds to various temperature conditions to improve seed germination. In addition, we examined *G*. *triflora* var. *japonica* seeds by studying the effects of cold stratification, GA_3_, and KNO_3_ on the dormancy release and seed germination characteristics under laboratory conditions and investigated a suitable germination protocol to confirm the feasibility of its practical application for the establishment of a mass production system.

## 2. Results

### 2.1. Water Imbibition Test

**Figure 1:**
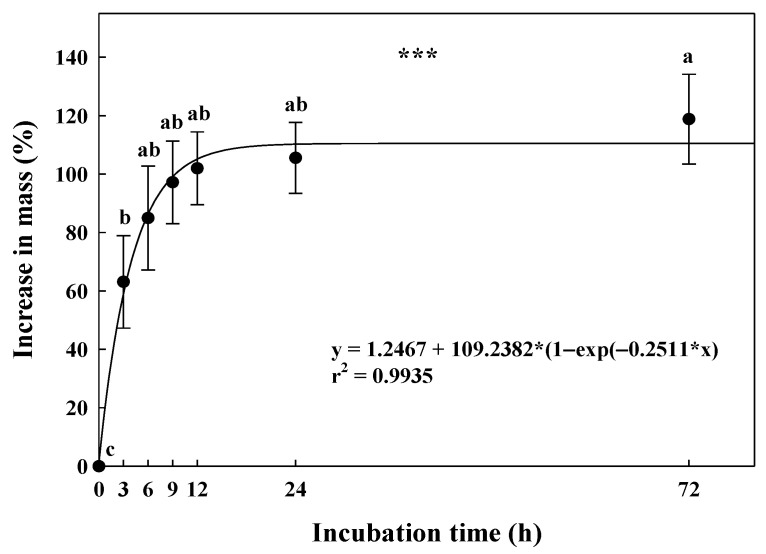
Changes in the mass of the *Gentiana triflora* var. *japonica* (Kusn.) H. Hara seeds during water incubation. The seeds were incubated at room temperature (approximately 25 ± 2 °C) on filter paper moistened with distilled water for 24 h. Vertical bars represent the standard deviation from the mean (*n* = 4). Different letters in the same column indicate significant differences based on Tukey’s multiple range test (*p* ≤ 0.05). *** Significant at *p* ≤ 0.001.

Water absorption by the *G*. *triflora* var. *japonica* seeds immediately increased their weight by 63.1 ± 15.8% compared to the initial mass after 3 h. In addition, 72 h after the immersion in distilled water, the seeds reached the saturated state at 118.8 ± 15.4% (Figure 1).

### 2.2. Seed Morphology and Embryo Growth

**Figure 2:**
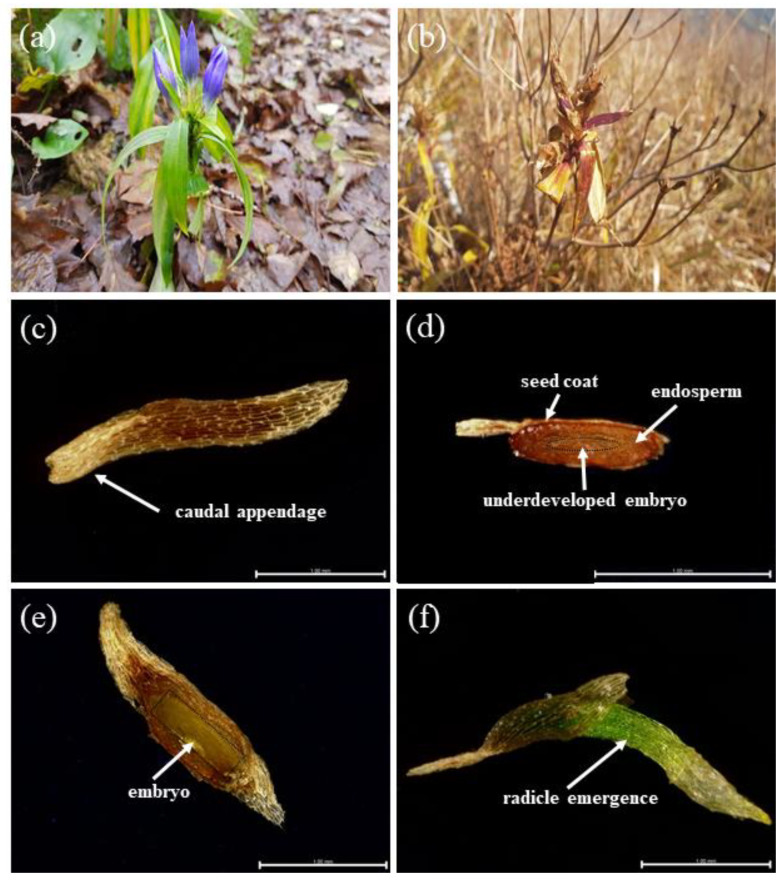
Flowers (**a**), capsules (**b**), seed (**c**), underdeveloped embryo at dispersal (**d**), fully developed embryo just before germination (**e**), and radicle emergence (**f**) of *Gentiana triflora* var. *japonica* (Kusn.) H. Hara (scale bar = 1 mm).

**Figure 3:**
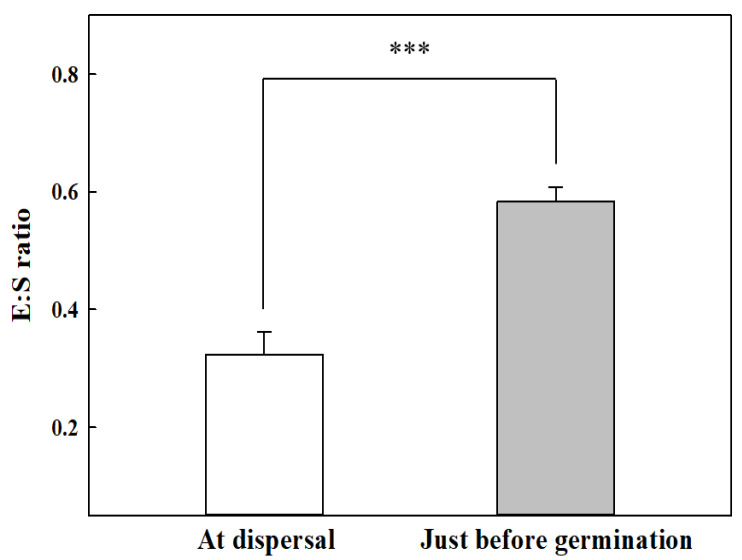
Embryo/seed (E:S) ratios in the seeds of *Gentiana triflora* var. *japonica* (Kusn.) H. Hara at seed dispersal and just before germination. Vertical bars represent the standard deviation from the mean (*n* = 10). Each E:S ratio at dispersal and just before germination was compared using a paired *t*-test. *** Significant at *p* ≤ 0.001.

The light-blue flowers of *G*. *triflora* var. *japonica* open in July and August in Korea, and one to five flowers occur at the upper leaf axil or at the tip (Figure 2A). In the case of fruits, the capsule types were composed of multi-core skin (Figure 2B). The seeds had caudal appendages at the time of dispersal, and the length and width of the seeds was less than 1 mm (Figure 2C). The seeds of *G*. *triflora* var. *japonica* had underdeveloped embryos at dispersal (Figure 2D). However, seed embryos were fully developed just before germination, and the radicle was protruded (Figure 2E,F). The embryo/seed (E:S) ratios at dispersal and just before germination were 0.32 ± 0.04 and 0.58 ± 0.03 mm, respectively (Figure 3).

### 2.3. Temperature Regime Treatments

**Figure 4:**
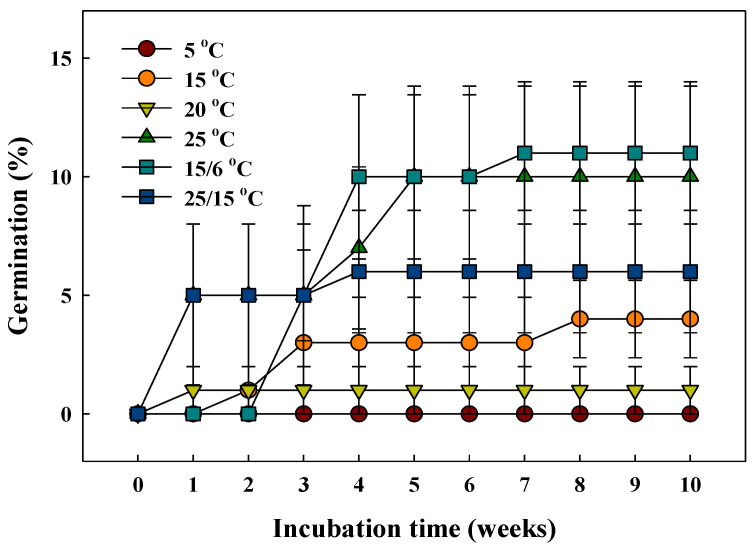
Cumulative germination of the *Gentiana triflora* var. *japonica* (Kusn.) H. Hara seeds in six temperature regimes (5, 15, 20, 25, 15/6, and 25/15 °C). Vertical bars represent the standard deviation from the mean (*n* = 4).

Figure 4 shows that the cumulative germination was influenced by temperature treatments used to release dormancy and determine the optimal temperature conditions for the germination of *G*. *triflora* var. *japonica* seeds. The average germination of all the temperature treatment groups during the 10-week incubation period was less than 12.0%. In the constant temperature groups at 5, 15, and 20 °C, the germination was remarkably lower than 5.0% over the 10 weeks. When the seeds were incubated at the alternating temperatures of 15/6 °C, the germination was 11.0 ± 3.0% for 7 weeks.

### 2.4. Cold Stratification Period

**Figure 5:**
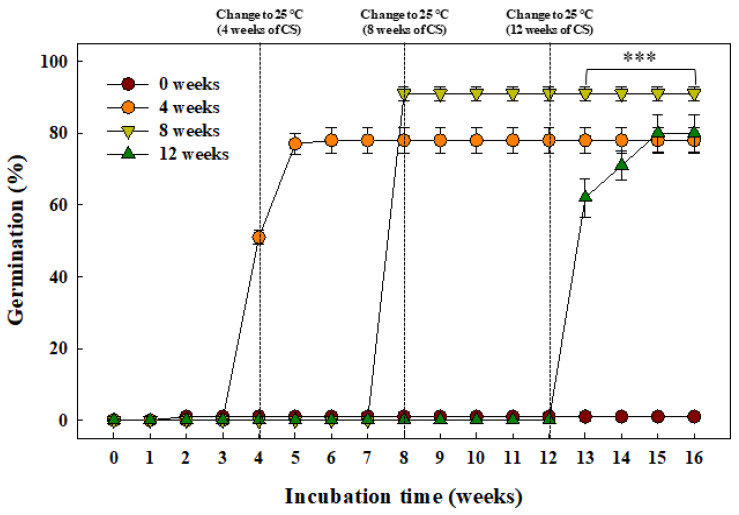
Cumulative germination of the *Gentiana triflora* var. *japonica* (Kusn.) H. Hara seeds treated with cold stratification (0, 4, 8, or 12 weeks). Vertical bars represent the standard deviation from the mean (*n* = 4). *** Significant at *p* ≤ 0.001.

The cumulative germination of the *G*. *triflora* var. *japonica* seeds, as influenced by the cold stratification period (0, 4, 8, or 12 weeks), is shown in Figure 5. The control seeds without cold stratification (0 weeks) did not germinate within 16 weeks. However, ≥78.0% of the seeds from the other treatment groups germinated after cold stratification for 4, 8, or 12 weeks. In particular, the germination was greater than 90.0% ± 1.9% at 16 weeks in the group with cold stratification for 8 weeks, which was significantly higher than that observed for the other treatment groups.

### 2.5. Promotion of Germination by GA_3_ and KNO_3_

**Figure 6:**
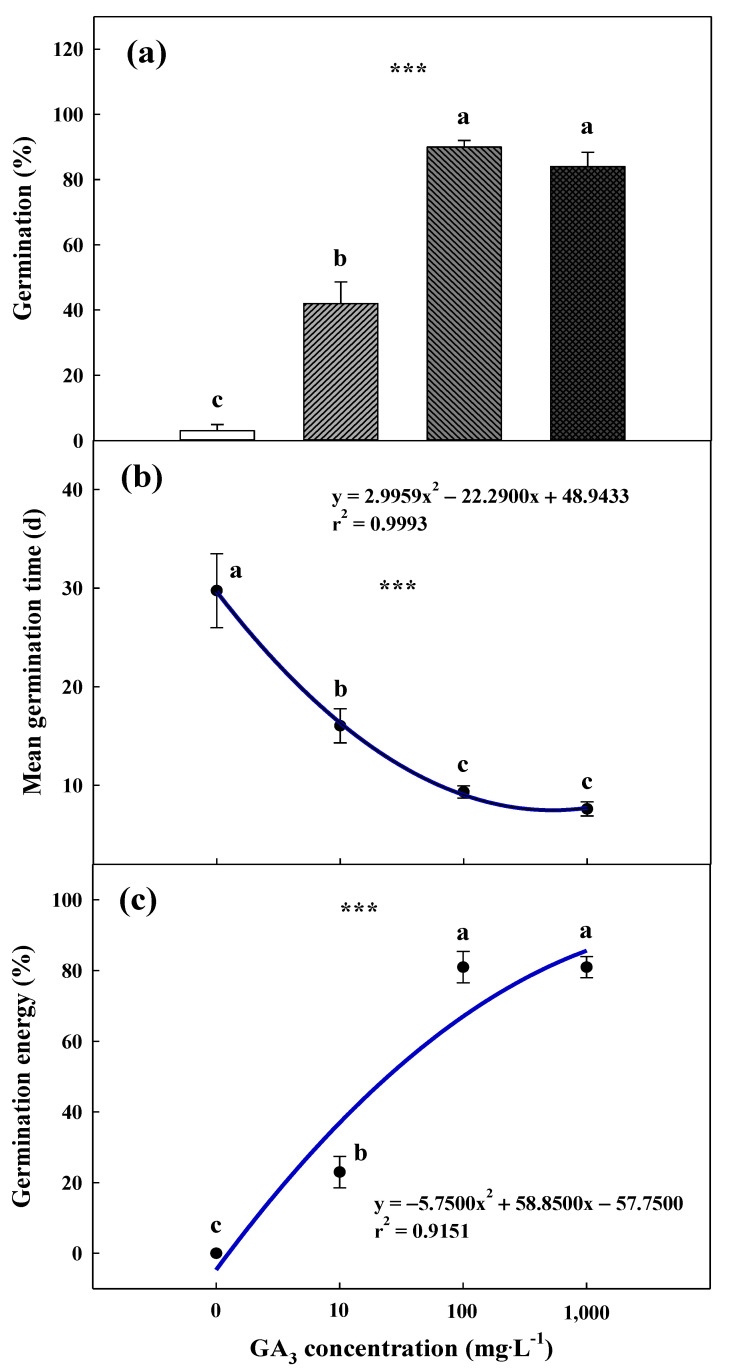
Germination percentage (**a**) and the mean germination time (**b**) of the *Gentiana triflora* var. *japonica* (Kusn.) H. Hara seeds pretreated with GA_3_ (0, 10, 100, or 1000 mg∙L^−1^) for 30 days. Germination energy (**c**) of the *Gentiana triflora* var. *japonica* (Kusn.) H. Hara seeds pretreated with GA_3_ (0, 10, 100, or 1000 mg∙L^−1^) for 7 days. Vertical bars represent the standard deviation from the mean (*n* = 4). Different letters in the same column indicate significant differences based on Tukey’s multiple range test (*p* ≤ 0.05). *** Significant at *p* ≤ 0.001.

**Figure 7:**
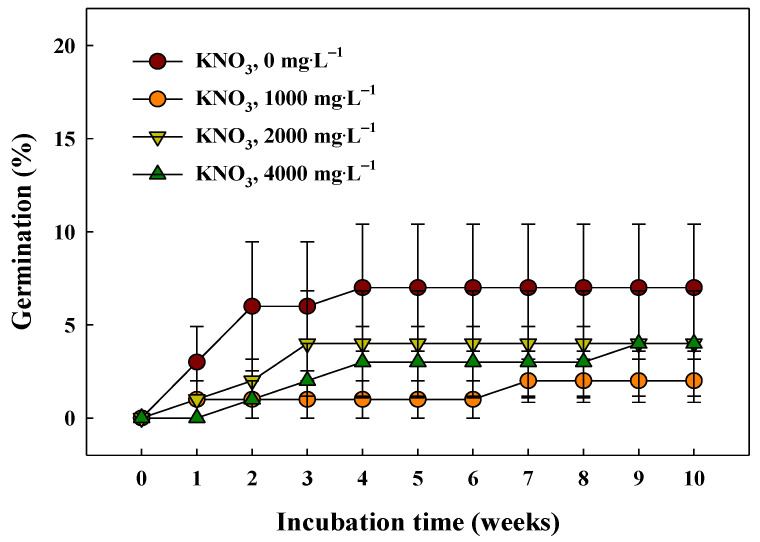
Cumulative germination of the *Gentiana triflora* var. *japonica* (Kusn.) H. Hara seeds pretreated with KNO_3_ (0, 1000, 2000, or 4000 mg∙L^−1^). Vertical bars represent the standard deviation from the mean (*n* = 4).

Germination of the *G*. *triflora* var. *japonica* seeds was improved following pretreatment with a GA_3_ solution (Figure 6A). The final germination of the seeds pretreated with 100 and 1000 mg·L^−1^ GA_3_ was 90.0 ± 2.0% and 84.0 ± 4.3%, respectively, with the mean germination time (MGT) of 9.3 ± 0.6 and 7.6 ± 0.7 days, respectively, which was significantly higher than the MGT in case of the other treatments (Figure 6B). Furthermore, the germination energy at 0–7 days was significantly increased with 100 and 1000 mg·L^−1^ GA_3_ (Figure 6C). However, germination was not significantly affected by the KNO_3_ concentration, indicating that osmotic priming by applying KNO_3_ was ineffective. In all the treatment groups, the cumulative germination did not exceed 10.0% until 10 weeks (Figure 7).

## 3. Discussion

### 3.1. Water Imbibition Test

Deficient water imbibition by intact seeds due to the seed coat or pericarp is a characteristic feature of PY dormancy [3]. From the ecological point of view, PY dormancy of seeds with a thick coat or pericarp can be a strategy to adapt to an unfavorable environment and germinate at an appropriate time for the plant life cycle [33]. Seeds with PY dormancy can release dormancy and germinate through various mechanical or chemical treatments that damage the seed coat or pericarp, such as dipping in boiling water, dry heating, and treating with sulfuric acid [16,34]. If the water absorption rate is less than 20.0% of the initial seed weight after 12 h of incubation under laboratory conditions, then the seed is classified as water-impermeable [35]. In this study, the seed mass increased by more than 100.0% after 12 h of immersion in distilled water (Figure 1). Consequently, the seeds of *G*. *triflora* var. *japonica* imbibed readily, suggesting that the species does not exhibit PY dormancy.

### 3.2. Seed Morphology, Embryo Growth, and Temperature Regime Treatments

The seed embryos of *G*. *triflora* var. *japonica* were underdeveloped at the time of dispersal and significantly increased in length just before germination (Figure 2B and Figure 3). According to Vandelook et al. [36], seed size and embryo length differ even within the same species, and the E:S ratio clearly represents the relative size of the embryos to the length of the seed. If the E:S ratio is >0.5, the embryo morphology is considered to be fully developed linear [37]. Seed dormancy with an undifferentiated or underdeveloped embryo can be classified as MD or MPD, which requires sufficient time for embryo development [3,38]. Generally, an underdeveloped embryo requires time to grow to the critical length for radicle protrusion, and Baskin and Baskin [20] reported that in seeds with MD dormancy, embryo development and germination occur within approximately 30 days under the optimal environmental conditions. The optimal germination temperature for each plant differs and is highly associated with the native habitat. Wild plant seeds native to temperate environments are known to have optimal germination temperatures between 24.0 °C and 30.0 °C [39]. In this study, however, the seeds had MPD, as evidenced by poor germination of less than 12.0% by 10 weeks in the temperature regime groups (Figure 4), which is common in wild plant seeds during germination experiments. MPD is subdivided into eight levels according to the following distinctions: (i) the optimal temperature to break dormancy and promote germination, (ii) the optimal temperature during embryo development, and (iii) the application of GA to break seed dormancy [20,40]. Specifically, it is divided into simple and complex MPD types, which are necessary at relatively high temperatures (≥15 °C) and low temperatures (0–10 °C) at the time of embryo growth. Therefore, simple and complex types are further subdivided into deep, intermediate, and non-deep dormancy based on the level of PD dormancy [20,38]. Breaking of MPD and acceleration of germination commonly utilize exposure to alternating temperatures and/or application of GA solutions.

### 3.3. Cold Stratification Period

Dormancy breaking and germination were examined during cold stratification (Figure 5). Germination was significantly higher in the cold stratification group than in the control group, and it significantly improved during the 8-week period after cold stratification in the other treatment groups. Chilling requirements play an essential role in breaking dormancy in many species whose seeds have MPD [20]. In *Arabidopsis*, cold stratification requires the activation of GA biosynthesis in intact seeds for germination [41]. These phenomena break seed dormancy by antagonizing phytohormones inside the seeds and are highly related with the expression of many genes involved in seed germination. Cold stratification has been reported to improve seed germination in some New Zealand species of the Gentianaceae [42]. However, there are differences in the duration of cold stratification for breaking dormancy between species of the same genus or family plant groups. In this study, 4–8-week cold stratification periods were determined as the optimal duration for dormancy release and embryo development in *G*. *triflora* var. *japonica* seeds.

### 3.4. Promoting Germination with GA_3_ and KNO_3_

In a previous study, dormancy breaking and germination were improved by applying GA_3_ to *Gentiana* species; this has also been observed in numerous other plant species [22,43,44,45,46]. Similarly, in this study, the final germination was enhanced by the application of 100 and 1000 mg∙L^−1^ exogenous GA_3_ at 90.0 ± 2.0% and 84.0 ± 4.3%, respectively (Figure 6A). Moreover, the MGT and germination energy (GE) values were improved following the application of GA_3_ at the concentrations of 100 and 1000 mg∙L^−1^ (Figure 6B,C). In a previous study, GA_3_ application was more effective at 1000 mg∙L^−1^ than at 50 and 100 mg∙L^−1^ and positively affected the percentage and speed of germination in *G*. *lutea* L. var. *aurantiaca* seeds [43]. In addition, 250 mg∙L^−1^ GA_3_ remarkably influenced the germination percentage compared to the results of 3-month cold stratification in *G*. *lutea* L. subsp. *lutea* seeds [22]. Endogenous phytohormones, especially GAs and abscisic acid (ABA), are known to regulate seed dormancy and germination. In PD-type dormancy, especially at non-deep levels, seed germination is regulated by quantitative changes and sensitivity to GAs and ABA [16,20]. Application of GAs promotes, the production of enzymes that hydrolyze cell walls after seed maturation, thereby accelerating radicle protrusion by decomposing the endosperm. In contrast, ABA suppresses GA synthesis and seed germination during dormancy release [47,48]. For this reason, the balance between GAs and ABA regulates the initiation, maintenance, and termination of seed dormancy. 

Osmotic priming treatments (such as KNO_3_, KCl, and NH_4_NO_3_) are large-scale pre-treatments used for commercial propagation of plants to promote effective and uniform germination, and their use is expanding to wild plant seeds [49]. Among them, KNO_3_ is most often used as an osmotic regulator; specifically, nitrate is absorbed into the seeds and activates antioxidant enzymes and seed embryo metabolism [50]. In addition, the absorbed nitrate is used as a nutrient, which affects the regulation of GA and ABA involved in seed germination [51,52]. Millaku et al. [44] determined that the seeds of *G*. *lutea* L. had higher germination when pre-treated with a mixed solution of 1000 mg∙L^−1^ GA_3_ and 0.1% KNO_3_. Furthermore, the germination of wild plant seeds of *Maesa japonica*, *Astilboides tabularis* (Hemsl.) Engl., and *Cicuta virosa* L. was increased by the KNO_3_ priming treatment at high concentrations [23,46,53]. Therefore, germination and embryo development may be enhanced by priming treatments, although the sensitivity varies with regard to concentration, treatment time, chemical material type, and plant species. However, in this study, pretreatments with KNO_3_ at different concentrations did not affect the cumulative germination of *G*. *triflora* var. *japonica* (Figure 7). Further studies using other priming materials and combinations of GAs are required to enhance the germination and uniformity of *G*. *triflora* var. *japonica* seeds and establish a mass propagation program.

## 4. Materials and Methods

### 4.1. Seed Preparation

The mature seeds of *G*. *triflora* var. *japonica* were collected on 24 October 2020 from plants growing at Mt. Sobaeksan, Danyang, Korea. After the seeds were cleaned, they were examined to determine seed characteristics. The seed length, seed width, and weight of 1000 seeds were 0.95 ± 0.04 mm, 0.38 ± 0.01 mm, and 0.0434 ± 0.002 mg, respectively. After the seeds were dried for 1–2 weeks, they were sealed in a plastic bag stored at 4 °C until the beginning of the experiment on 11 March 2021.

### 4.2. Water Imbibition Test

The ability of the seeds to imbibe water under laboratory conditions (approximately 25 ± 2 °C, room humidity (RH) of 40%–50%) was determined. Four replicates of 100 seeds were initially weighed using an electronic balance (ML204/01, Mettler Toledo, Columbus, OH, USA). Subsequently, the *G*. *triflora* var. *japonica* seeds from each replicate were individually placed on two layers of filter paper (Whatman No. 2, Toyo Roshi Kaisha, Ltd., Tokyo, Japan) moistened with distilled water in 90 × 15 mm plastic Petri dishes (SPL Life Sciences Co., Ltd., Pocheon, Korea). The fresh weight of the *G*. *triflora* var. *japonica* seeds was measured after 3, 6, 9, 12, 24, and 72 h of incubation, and the water uptake by seeds was calculated using the water uptake formula detailed by Baskin and Baskin [20]:

% W_s_ = [(W_i_−W_d_/W_d_)] × 100 (1)

where W_s_ is the increase in seed mass, W_i_ is the seed mass after a given interval of imbibition, and W_d_ is the initial seed mass at 0 h.

### 4.3. Seed Morphology and Embryo Growth

To identify MD dormancy, *G*. *triflora* var. *japonica* seeds were incubated at 25 °C, and then the measurement of seed morphology was carried out from 11 March to 24 May 2021. Ten seeds were cut along the major or minor axis using a razor blade (stainless blade, Dorco, Seoul, Korea), and then the cross-section was measured under a digital microscope (DVM6, Leica Microsystems GmbH, Wetzlar, Germany). The increase in embryo length was measured at dispersal and just before germination. The ratio of embryo length to seed length (E:S ratio) was calculated using the formula detailed by Vandelook et al. [36].

E:S ratio = seed length/embryo length (2)

### 4.4. Temperature Regime Treatments

To identify the optimal temperature for germination, the seeds were incubated at 5, 15, 20, 25, 15/6, and 25/15 °C with a photoperiod of 12/12 h (light/dark) in a growth chamber (TGC-130H, Espec Mic Corp., Aichi, Japan). Light intensity during the daytime across all temperature regimes was 40 ± 10 μmol∙m^−2^∙s^−1^ photosynthetic photon flux density (PPFD) using fluorescent lamps. Four replicates of 25 seeds were placed separately on a 1% agar (Agar, Sigma-Aldrich, St. Louis, MO, USA) medium in 60 × 15 mm plastic Petri dishes (SPL Life Sciences Co., Ltd., Pocheon, Korea), which were sealed with a parafilm (PM-996, Bemis Company Inc., Neenah, WI, USA) during incubation.

### 4.5. Cold Stratification Period

To investigate the response to cold stratification for germination, the seeds were incubated for 0, 4, 8, or 12 weeks at 5 °C. After each cold stratification period, the seeds were transferred to a growth chamber (25 °C with a photoperiod of 12/12 h (light/dark)). Light intensity during the daytime across all the temperature regimes was 40 ± 10 μmol∙m^−2^∙s^−1^ PPFD using fluorescent lamps. Four replicates of 25 seeds were each placed separately on a 1% agar medium in 60 × 15 mm plastic Petri dishes, which were sealed with a parafilm during incubation.

### 4.6. Promoting Germination with GA_3_ and KNO_3_

Four different concentrations of a GA_3_ (≥ 90%, Sigma-Aldrich, St. Louis, MO, USA) solution (0 (control), 10, 100, or 1000 mg∙L^−1^) and of a potassium nitrate (KNO_3_, 99.0%; Daejung Chemical Co., Ltd., Siheung, Korea) solution (0 (control), 1000, 2000, or 4000 mg∙L^−1^) were applied to promote germination. The seeds were soaked in the various solutions for 24 h under laboratory conditions (approximately 25 ± 2 °C, RH of 40%–50%). The control groups were soaked in distilled water for 24 h. After the pretreatment with GA_3_ and KNO_3_, the seeds were washed five times with distilled water, and germination characteristics were observed at 25 °C in a growth chamber (12/12 h light/dark photoperiod with fluorescent lamps at 40 ± 10 μmol∙m^−2^∙s^−1^ PPFD). Four replicates of 25 seeds were each placed separately on a 1% agar medium in 60 × 15 mm plastic Petri dishes, which were sealed with a parafilm during incubation.

To evaluate the GA_3_ pretreatment, the seed germination stage, germination (G), MGT, and GE of the *G*. *triflora* var. *japonica* seeds were calculated as follows [42,54]:

% G = G_30_/N × 100 (3)

MGT (days) = ∑ (T × S)/∑ S (4)

% GE = G_7_/N × 100 (5)

where N is the total number of seeds, T is the time in days from day 1 to the final day of the germination test, and S is the total number of germinated seeds on day T. G_7_ and G_30_ are the total numbers of seeds germinated on days 7 and 30, respectively, after sowing.

### 4.7. Data Collection and Statistical Analysis

The number of germinated seeds was counted for 2–3 days. Each seed was considered to have germinated when the radicle emergence reached at least 2 mm. Statistical analyses were performed using the statistical analysis system (SAS 9.4, SAS Institute Inc., Cary, NC, USA). The differences between the E:S ratios of seeds at dispersal and immediately before germination were assessed using paired *t*-tests. The germination experiment results were subjected to analysis of variance and Tukey’s multiple range tests (*p* ≤ 0.05). Regression and graphing were performed using SigmaPlot 12.0 (Systat Software Inc., San Jose, CA, USA).

## 5. Conclusions

This study concluded that the *G*. *triflora* var. *japonica* seeds had intact seed coats that were permeable to water absorption and did not exhibit PY dormancy. In addition, the seeds were detached in an immature embryo state and showed MD dormancy that required embryo growth until just before germination, similar to the Gentianaceae family [16,18]. Furthermore, in the temperature treatments, the cumulative germination did not reach even 15.0% within 30 days; therefore, the seeds were judged to have MPD dormancy. However, no seeds were affected by osmotic priming (KNO_3_ treatment). In conclusion, *G*. *triflora* var. *japonica* seeds have a non-deep simple MPD, which can be released by cold stratification and pretreatment with GA_3_ to completely break dormancy and enhance germination. Considering the decrease in MGT and dormancy breaking of *G*. *triflora* var. *japonica* seeds, pretreatment with 100 mg∙L^−1^ GA_3_ is an effective method of releasing dormancy. In addition, cold stratification for 4–8 weeks could serve as an alternative to GA_3_ for seed germination. This knowledge aids in clarifying the ecophysiological mechanisms under different environmental conditions and in developing useful methods for obtaining *G*. *triflora* var. *japonica* seedlings in a mass propagation setting.

## Data Availability

Not applicable.
